# Automatic synchrotron tomographic alignment schemes based on genetic algorithms and human-in-the-loop software

**DOI:** 10.1107/S1600577522011067

**Published:** 2023-01-01

**Authors:** Zhen Zhang, Xiaoxue Bi, Pengcheng Li, Chenglong Zhang, Yiming Yang, Yu Liu, Gang Chen, Yuhui Dong, Gongfa Liu, Yi Zhang

**Affiliations:** a National Synchrotron Radiation Laboratory, University of Science and Technology of China, Hefei, Anhui 230029, People’s Republic of China; b University of Chinese Academy of Sciences, Beijing 100049, People’s Republic of China; c Beijing Synchrotron Radiation Facility, Institute of High Energy Physics, Chinese Academy of Sciences, Beijing 100049, People’s Republic of China; Australian Synchrotron, Australia

**Keywords:** scanning tomography, rotation-axis alignment, sample alignment, genetic algorithms, human-in-the-loop, computed tomography, X-ray microscopy

## Abstract

Tomography imaging methods at synchrotron light sources keep evolving, pushing multi-modal characterization capabilities at high spatial and temporal resolutions. To achieve this goal, small probe size and multi-dimensional scanning schemes are utilized more often in the beamlines, leading to rising complexities and challenges in the experimental setup process. To avoid spending a significant amount of human effort and beam time on aligning the X-ray probe, sample and detector for data acquisition, most attention has been drawn to realigning the systems at the data processing stages. However, post-processing cannot correct everything, and is not time efficient. Here we present automatic alignment schemes of the rotational axis and sample pre- and during the data acquisition process using a software approach which combines the advantages of genetic algorithms and human intelligence. Our approach shows excellent sub-pixel alignment efficiency for both tasks in a short time, and therefore holds great potential for application in the data acquisition systems of future scanning tomography experiments.

## Abstract 2

A highly automatic alignment scheme is proposed to address the pressing challenge in tomographic alignment of future scanning tomography experiments. The results show that the proposed method exhibits excellent sub-pixel alignment accuracy and high time efficiency.

## 1. Introduction

**Figure 1:**
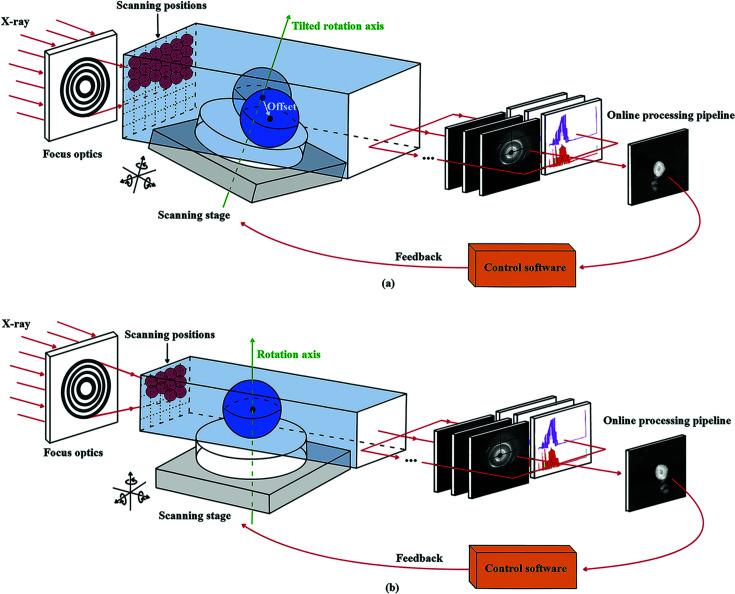
A larger scanning area is required due to misalignment (*a*) of the scanning tomography setup compared with perfectly aligned (*b*) systems.

In order to image a larger sample volume while retaining high spatial resolution, scanning tomography methods are becoming increasingly popular (Rawson *et al.*, 2020 ▸; Sakdinawat & Attwood, 2010 ▸; Withers *et al.*, 2021 ▸; Withers, 2007 ▸) at synchrotrons. Scanning tomography experiments usually involve multi-dimensional scans across the sample coupled with multimodal characterizations using X-ray beams with small spot sizes. Scientists seek to incorporate various synchrotron techniques like X-ray fluorescence (XRF), X-ray diffraction (XRD), X-ray absorption near-edge structure (XANES), ptychography, *etc.* in scanning tomography experiments to correlate the local chemical, phase, strain, oxidation states and other functional properties with the structure of advanced materials (Liu *et al.*, 2021 ▸; Zhao *et al.*, 2018 ▸; Nguyen *et al.*, 2021 ▸; Vojtová *et al.*, 2019 ▸; Kodama *et al.*, 2021 ▸; Müller *et al.*, 2021 ▸), hence providing a deeper understanding of structure–function relationships. For experiments carried out in micro- or nanoprobe beamlines, the amount of time necessary to collect a full dataset composed of hundreds of thousands of scan points is quite significant (Sasov, 2004 ▸). Therefore, the majority of the experiments performed at current third-generation light sources are limited to static characterization. At present, the main reason for the long scan times is the relatively low beam intensities. While diffraction-limited storage rings will bring game-changing brilliant sources to speed up the acquisition process and potentially meet the demand of dynamic or operando studies, the inconvenience of achieving a fine alignment between the X-ray probe and the examined sample (Fig. 1 ▸) may also be a major limiting factor in accelerating the whole acquisition process.

Generally, a 2D raster scan will be carried out at each projection and then the process is identical to the full-field imaging methods with the sample stage rotating to different angles. To satisfy the tomography reconstruction requirements, the raster scan region needs to be capable of covering the sample area at all projections. While for full-field tomography the sample is not necessarily required to be precisely aligned to the center of the field of view, in scanning tomography experiments a bad alignment of the sample with the rotation center means an enlarged area needs to be scanned at each projection, thereby causing unnecessary time loss. Unlike the straightforward alignment process directly from projection images in full-field imaging (Ma *et al.*, 2018 ▸), the alignment of the sample with respect to the rotation center in scanning tomography is usually far more complicated. Currently scientists are mainly relying on in-line microscopes to conduct coarse alignment at beamlines. However, the alignment accuracy using microscopes will not meet future scanning tomography experimental demands as the probe size reaches the nanometer range. A precise alignment should be based on the functional projection images acquired from real-time analysis of XRF, XRD and ptychography signals *etc*. Due to the complex alignment procedure and poor resolution of the composed projection image, a more automated and intelligent alignment method needs to be developed to cater to the fine alignment requirements of future scanning tomography experiments.

Although adding a layer of complexity, the obtained XRF, XRD and ptychography data from each projection can be automatically processed in real time using custom processing pipelines. The remaining alignment process of the scanning tomography will be based on 2D projection images, which shares great similarity with full-field tomography. Hence, alignment methods developed for full-field tomography can also be applied to scanning tomography experiments. In general, the tomographic alignment includes rotation-axis alignment and sample alignment with respect to the center of rotation (CoR) (Dong *et al.*, 2013 ▸; Donath *et al.*, 2006 ▸; Yu *et al.*, 2019 ▸; Yang *et al.*, 2015 ▸). For both scanning and full-field tomography, the rotation axis needs to be precisely aligned to be perpendicular to the incoming beam direction and parallel to the detector plane. Jun & Yoon (2017 ▸) proposed a method to correct the tilt error of the rotation axis using the center of attenuation and its sinograms. Cheng *et al.* (2018 ▸) minimized the total deviation of the reconstructed image to correct problems such as errors resulting from incorrectly determined rotational axes and projection angles. However, comparing with other factors, the quality degradation of the reconstructed structure due to the misalignment of the rotation axis will be more difficult to compensate by reconstruction algorithms. Therefore, the rotation axis should be well aligned pre-acquisition. Once the rotation axis alignment is completed, the sample alignment procedures are ready to be initiated. In reality, the sample alignments with respect to the CoR are usually done at the post-acquisition stage. A wide range of post-acquisition sample alignment schemes based on image processing techniques (McEnvoy, 2007 ▸; Matula *et al.*, 2003 ▸; Amat *et al.*, 2008 ▸; Cheng *et al.*, 2014 ▸; Pan *et al.*, 2012 ▸; Robertson *et al.*, 2015 ▸; Sorzano *et al.*, 2020 ▸) or iterative reconstruction-reprojection (Nassi *et al.*, 1982 ▸; Ollinger, 1990 ▸; Wang *et al.*, 2000 ▸; Li *et al.*, 2020 ▸) methods have been developed and have shown great effects. In particular, Vo *et al.* (2014 ▸) proposed a sample alignment scheme based on Fourier space sinograms. Yang *et al.* (2017 ▸) used convolutional neural networks to reduce the horizontal drift problem caused by aspects such as instrument instability.

All the above methods show well aligned results in their respective application scenarios; however, most of them focus on the post-processing stage of tomography experiments, and these alignment algorithms cannot correct everything and are not time efficient. Therefore, in contrast to the above alignment schemes that concentrate on the post-acquisition process, we report here an alignment method in the pre-acquisition process that aligns the rotation axis first and then the sample. The method divides the workflow of rotational-axis alignment and sample alignment into two steps – coarse alignment and fine alignment – and provides a graphical user interface (GUI) and manual intervention to assist the alignment procedure. In particular, the algorithm used for coarse alignment is based on a physical model and the process is usually very fast. To reach a more accurate alignment, a genetic algorithm (GA) (Kihm & Lyons, 1996 ▸) is introduced which can optimize the motor position in the global scope to realize an automatic alignment. The GA is a global optimization algorithm which ensures the final results are globally optimized. Moreover, for a subjective function to be optimized by the GA, its gradient information and mathematical model are not required. The only necessary information is the output corresponding to a certain input. These facts lead to the conclusion that a GA-based optimization can be performed at any sample without parameter modification. Virtual tomography experiments were conducted for the algorithm verification and software validation; the results demonstrate that our method can achieve sub-pixel alignment accuracy. Human intervention through the designed GUI has also shown significant importance in facilitating the alignment process. Together, our method has demonstrated great application potential for enhancing the data acquisition efficiency of future synchrotron scanning tomography experiments.

## 2. Methods

### 2.1. Virtual tomography experiment

The in-house-developed *Virtual Beamline Studio* (*VBS*) provides a test bed for verification of the proposed algorithms in the paper. *VBS* was developed to simulate the future multi-modal and high-data-throughput experiments performed at the next-generation beamlines of the High Energy Photon Source (HEPS). The user can employ a certain combination of virtual detectors and motors to perform a virtual experiment in *Mamba* (Liu *et al.*, 2022 ▸). The simulated detectors and motors share the sample protocols with real devices in terms of control and data communication in the data acquisition software framework (*Mamba*) developed for HEPS. The only difference is, instead of reading data from chips, the virtual detectors read images which are stored in memory or generated from third-party simulation programs. The virtual experiment enables users to test and implement algorithms and software applications without a real experimental setup. To simulate tomography experiments, we developed a simulation model that generates projection images using reconstructed sample slices with defined measurement geometry (sample, beam and detector). In the procedure, this module performs rotation and integration operations on all reconstructed sample slices, and converts three-dimensional sample slices into a series of two-dimensional sample projection images according to the Radon transformation. The samples were simulated using the tomography dataset from Amat *et al.* (2008 ▸) and a XANES tomography dataset collected from Stanford Synchrotron Radiation Lightsource (SSRL). For the first set of walnut data, we selected the Feldkamp–Davis–Kress (FDK) reconstruction data of the first set of walnuts as the primary sample. This is a collection of computed tomography slices that are highly aligned with no artifacts present and well suitable for algorithm development and evaluation for other tasks. Furthermore, the second dataset is the one that we would face in our actual scanning tomography experiments which is of great help for the validation of our algorithm. The simulated motors read the predefined trajectories to mimic the scans in real experiments.

### 2.2. Rotation-axis alignment

**Figure 2:**
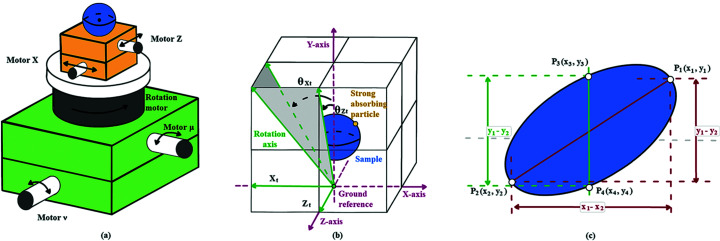
(*a*) Sample positioning stage. Motor *x* and motor *z* control the movement of the sample, the rotation motor controls the rotation of the sample, and motor μ and motor ν control the tilt of the rotation axis. (*b*) Rotary-axis tilt model. The tilt angle of the axis of rotation can be split into the superposition of two orthogonal vectors. (*c*) Physical model of the rotary axis coarse alignment. The adjustment angle of the axis of rotation is roughly calculated from the coordinates of the four feature points.

**Figure 3:**
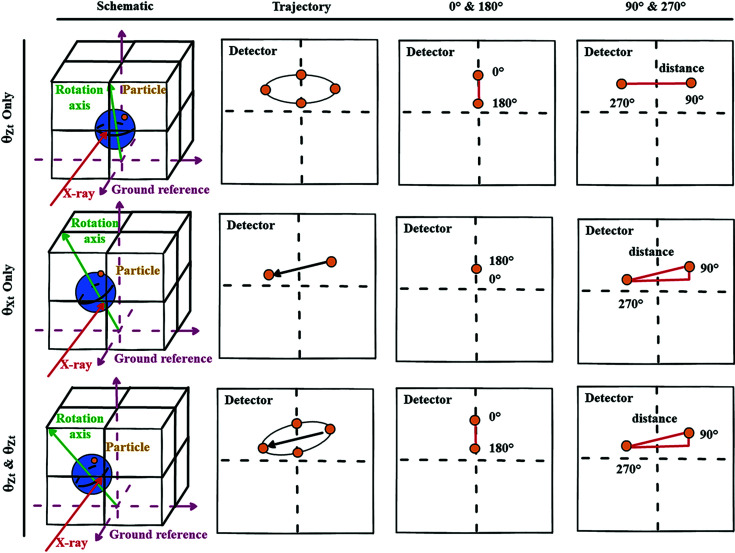
Particle trajectories under different rotation-axis misalignment situations.

During synchrotron experiments the rotation axis usually only needs to be realigned once for each beam time, and therefore users rarely use real samples to align the rotation axis but instead tend to use a specialized calibration sample with sharp features. For convenience, we use the walnut sample for both rotation-axis alignment and sample alignment tests. To facilitate the alignment of the rotation axis with respect to the detector, we randomly added a strong absorbing particle on the surface of the walnut sample. As shown in Fig. 2 ▸, the position of the sample can be represented by a 3D coordinate system, and the tilt of the rotation axis can be decomposed as a superposition of two orthogonal vectors *Z*_*t*_ and *X*_*t*_. For the effect caused by these two vectors (θ_*Zt*_ and θ_*Xt*_), we can align the rotation axis by adjusting motor μ and motor ν. It is necessary to note that in a real rotation-axis alignment scenario, the staff usually use a needle or other standard sample with one strong absorbing particle. Therefore, temporarily, here we only extract and track one strong absorbing particle of the walnut sample, which can be easily filtered out by setting a higher grayscale threshold to obtain its motion trajectory. Ideally, the trajectory of the particle will form a straight line after one turn of rotation without any tilt (θ_*Xt*_ = 0, θ_*Zt*_ = 0). As shown in Fig. 3 ▸, when θ_*Xt*_ = 0 and θ_*Zt*_ ≠ 0, the trajectory of the particle turns out to be an ellipse. When θ_*Xt*_ ≠ 0 and θ_*Zt*_ = 0, the trajectory of the particle forms a straight line at a certain angle to the horizontal direction, with its two endpoints collected from projection angles 90° and 270°. When θ_*Xt*_ ≠ 0 and θ_*Zt*_ ≠ 0, the trajectory of the particle will be a tilted ellipse, from which θ_*Xt*_ and θ_*Zt*_ can be calculated as

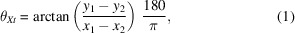




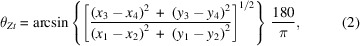

where *x*_1_, *x*_2_, *x*_3_ and *x*_4_ are the horizontal coordinate values of *P*_1_, *P*_2_, *P*_3_ and *P*_4_, and *y_1_*, *y*_2_, *y*_3_ and *y*_4_ are the vertical coordinates values of *P*_1_, *P_2_*, *P*_3_ and *P*_4_, respectively. *P*_1_ and *P*_2_ are the vertices of the major axis of the ellipse fitted to the trajectory of the feature particle, while *P*_3_ and *P*_4_ are the two points on the ellipse with the same *x*-coordinate of the midpoint of the minor axis.

**Figure 4:**
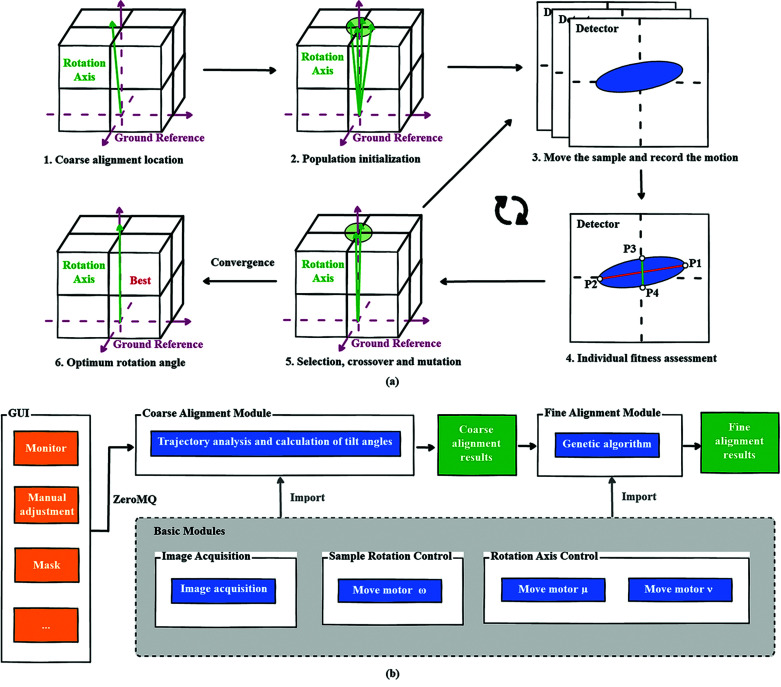
(*a*) The process of fine alignment of the rotation axis. Genetic-algorithm-based selection, crossover, mutation and inheritance processes for rotating-axis individuals. (*b*) Framework design of the rotation alignment software. The blue color indicates the rotation alignment algorithm and its associated functional modules, and the orange color indicates the GUI module.

A two-step alignment procedure is introduced for the rotation-axis alignment: coarse alignment and fine alignment, as shown in Fig. 4 ▸. For the coarse alignment, the tilt angles of motor μ and motor ν are corrected by equations (1) and (2). For the fine alignment of the rotation axis based on the GA, a series of individuals of random rotation-axis positions (motor μ and motor ν) are generated to form the initial population around the coarsely aligned rotation-axis position. The population fitness is assessed according to the trajectory characteristics of the strong absorbing particle. After several rounds of selection, crossover and mutation, the population finally reaches convergence and obtains the optimum adjustment angles. To avoid repetition in data acquisition, the trajectory information of new individuals in the previous generations will be stored for the new iteration. Typically, we set two adjustment accuracy modes to accommodate different adjustment needs: (*a*) 15 population iterations for a population of 20 individuals and (*b*) 15 population iterations for a population of 30 individuals. The number of population iterations and individuals are application-scenario-dependent input parameters for the alignment program. The program can also be terminated early by human intervention if the population has converged.

### 2.3. Sample alignment

To simulate the projection images for a misaligned sample with respect to the rotation axis, an offset was applied on the sample. In the virtual experiment, this offset is a combination of the offsets of the two sample motors (motor *x* and motor *z*), which we denote as *offset_x* and *offset_z*. The physical model for the coarse alignment is shown in Fig. S1 of the supporting information. During the coarse alignment of the sample, firstly we performed one coarse tomography scan and checked whether the sample was within the field of view (FoV) of the detector for all projections. If the sample was falling out of the FoV at certain projections, another scan was performed from 0° to 180° to find the projection angle ω_1_ where the sample was completely rotated back into the FoV and the angle ω_2_ where the center of the sample reached the center of the detector.

After that, we obtained the distance between the sample center and the detector center at the ω_1_ angle and denoted this distance as *distance_ω_1_*. Among them, the offsets of the two motors can be expressed as

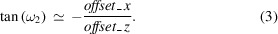

Meanwhile, *distance_ω_1_* can be expressed as the superposition of two offsets at angle ω_1_,

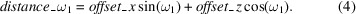

Here, according to the relationship of the offset and the reference angles, we can obtain *offset_x* and *offset_z* by equations (5) and (6),

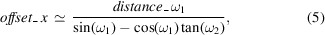




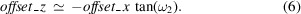

**Figure 5:**
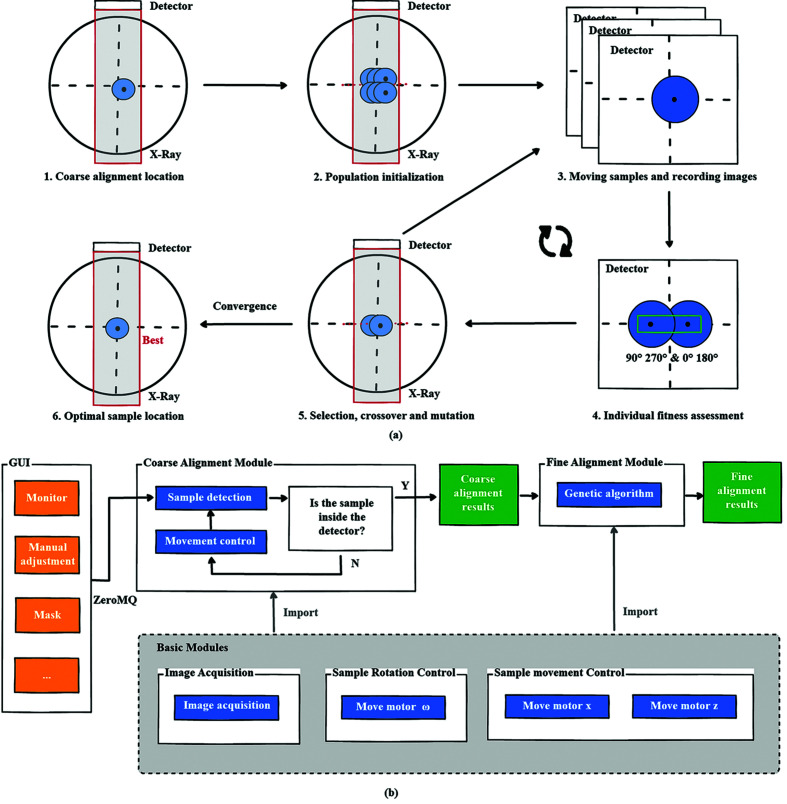
(*a*) The process of fine sample alignment. (*b*) Framework design of the sample alignment software. The blue color indicates the sample alignment algorithm and its associated functional modules, and the orange color indicates the GUI module.

The sample was then moved according to the calculated *offset_x* and *offset_z* for the coarse alignment. Following the coarse alignment, a fine alignment process triggered by genetic algorithm is required for further alignment work as shown in Fig. 5 ▸. Based on genetic algorithm, we use movement values of the two motors as the gene and the sum of the difference between the center of the sample at 90° and 270° and the difference between the center of the sample at 0° and 180° as the fitness evaluation metric. After the initial population has evolved through selection, crossover and mutation, the individual eventually converges, and that individual is the optimal distance to move for sample precision alignment. To speed up the program, the images acquired from motor positions which have been generated in the previous generations will be reused in the new iteration. In practice, the current position is not exactly accurate due to sample center calculations, movement errors, *etc*.

### 2.4. User interaction design

As shown in Figs. 5 ▸(*a*) and 5 ▸(*b*), the framework design of the software is mainly composed of two parts – the GUI and the backend server. Among them, the GUI has been developed based on *PyQt* and provides a number of advanced interfaces that enable users to monitor the alignment status and define parameters for the algorithms. The backend server integrates the coarse alignment module, the fine alignment module and some basic motion control modules. The GUI and backend server communicate through the *ZeroMQ* protocol.

## 3. Results

### 3.1. Rotation-axis alignment

**Figure 6:**
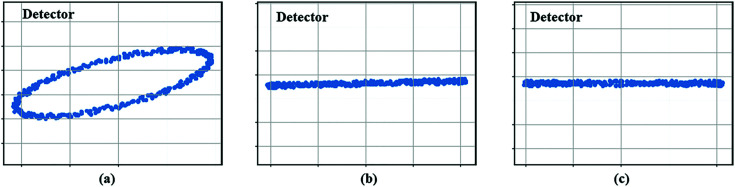
(*a*) Initial trajectory with tilted rotation axis. (*b*) Trajectory after coarse alignment. (*c*) Trajectory after fine alignment.

Currently, a large number of tomography beamlines rely on using calibration samples with sharp edges or gold particles to accomplish the rotation-axis alignment. Here we applied the rotation-axis alignment algorithm to a simulated rotational model with a gold particle. For demonstration, the rotation axis was initially defined as parallel to the vector with θ_*Zt*_ set to be 14.01° and θ_*Xt*_ set to be 14.01°, and the initial coordinate of the gold particle was set to [150, 81, 138]. In addition, a random noise error was added to the rotation axis to more realistically simulate the movement of the gold particle. The results of the complete axis alignment are shown in Figs. 6 ▸(*a*), 6(*b*) and 6(*c*). In the initial state, there were significant offsets in the movement trajectory of a gold particle at rotation angles of 0, 90, 180 and 270. After a coarse alignment of the rotation axis, a reduction in the offset was clearly observed at the four angles mentioned above. The rotational trajectory of the gold particle also transformed from a larger ellipse to a smaller ellipse with θ_*Zt*_ reduced to 1.43°. Then, after a fine alignment based on genetic algorithms, with the number of individuals within the population set at 20, with 48 genes per individual and 15 generations of evolution, both orthogonal tilt angles of the rotation axis finally converged to close to 0°. In theoretical terms, the rotation axis has been adjusted to an ideal position for subsequent sample alignment. In practice, the algorithm should be applicable for samples with arbitrary characteristic points such as gold particles, needle tips, *etc*. and the efficiency will also be limited by the accuracy of the motors and the pixel size of the detector. For a fine rotation-axis alignment, only 42 s was needed for the individuals within the population to be converged if the acquisition time for the projection images is not accounted for.

In a real scenario, the initial position of the gold particle can be very close to the rotation axis, which may raise potential difficulty for the algorithm. Therefore, to verify the robustness of the algorithm, we initialized a series of gold particles at different positions and compared their alignment results, as demonstrated in Fig. S2 of the supporting information. The effect of the alignment of the rotation axis is gradually affected as the distance between the gold particle and the rotation axis decreases. When the initial position was ahead of [0, 6, 0], the distance between the gold particle and the rotation axis was about two pixels at this time, and the rotation axis could be better calibrated. The orthogonal tilt angles of the rotation axis finally converged to close to 0° after fine alignment.

### 3.2. Sample alignment

**Figure 7:**
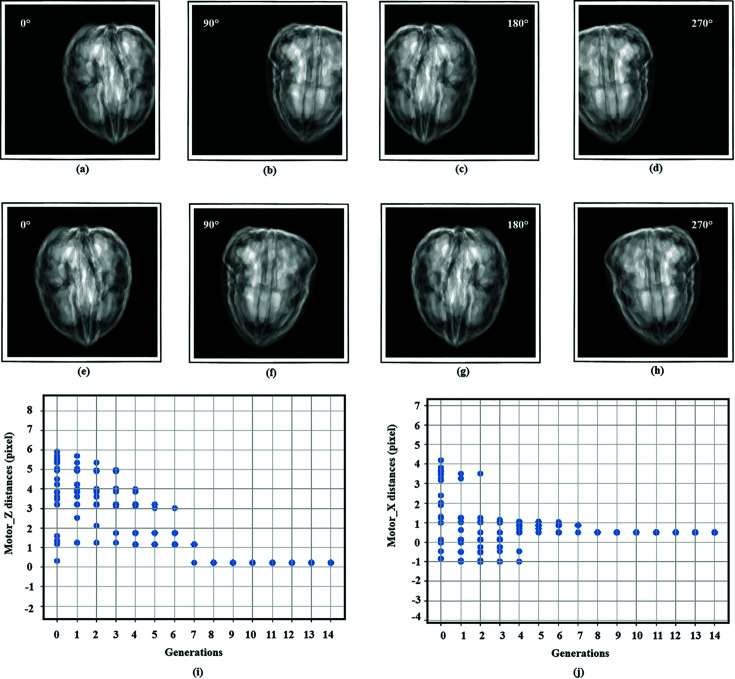
(*a*)–(*d*) Projection images of the sample before alignment. (*e*)–(*h*) Projection images after coarse alignment. (*i*)–(*j*) Sample offset in the *x* and *z* directions after each generation when the number of individuals in the population is set to 20 during the fine alignment procedure.

For the sample alignment, a coarse alignment procedure is necessary to move the sample completely into the FoV for all projections when the sample is too far away from the CoR. To test the coarse alignment module, we used the walnut dataset as the data source and set large offsets of the sample (159 and 104 pixels in the *x* and *z* directions, respectively) with respect to the CoR as the initial position. As shown in the projection images [Fig. 7 ▸(*a*) to 7(*h*)], the sample falls out of the FoV of the detector in certain projections. After the coarse alignment, it can be observed that the sample had been moved all the way into the imaging area of the detector. Until now there was still a two-pixels offset in the *x* direction and a three-pixels offset in the *z* direction for the following fine alignment. With the number of individuals within the population set to be 20, the offset was reduced to 0.51 (*x* direction) and 0.73 (*z* direction) pixel after fine alignment. The comparative results of the experiments are shown in Figs. 7 ▸(*i*) and 7 ▸(*j*). The time consumption of the fine alignment was only 50 s when not considering the simulation time to generate the projection images calculation, which in real experiments will be the sample rotation and image acquisition time. The results show that a very high accuracy alignment was achieved for the walnut sample at a short time cost.

**Figure 8:**
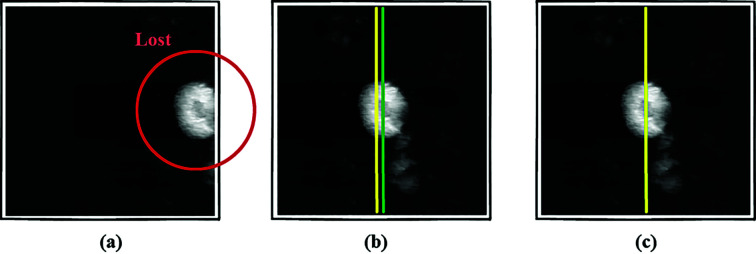
Algorithms validation using a XANES dataset.

Finally, to test the alignment efficacy of the scanning tomography experiment, we utilized poor-resolution data collected from a XANES experiment at SSRL. We set the initial *offset_x* to 178 pixels and *offset_z* to 117.5 pixels [Fig. 8 ▸(*a*)] and the alignment accuracy to the first level, and the entire alignment process took approximately 48 s. As shown in Fig. 8 ▸(*b*), at the end of the coarse alignment, the samples had an *offset_x* of 2.5 pixels and an *offset_z* of 3 pixels. At the end of the fine alignment [Fig. 8 ▸(*c*)], *offset_x* = 0.43 pixel and *offset_z* = 0.14 pixel, showing a better alignment.

### 3.3. Human-in-the-loop control software

**Figure 9:**
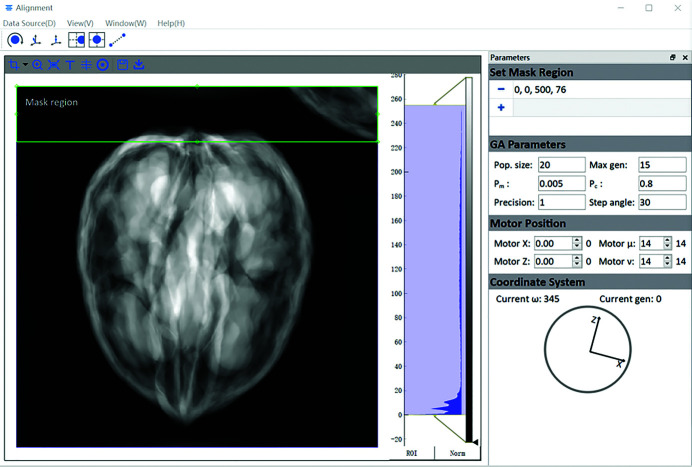
GUI of the alignment software.

An interactive GUI was developed to facilitate the alignment procedure. As shown in Fig. 9 ▸, the GUI not only provides visualization of the streaming projection images, current acquisition parameters, alignment progress information, *etc*. but also components for the users to set and change the alignment parameters. At the moment, the displacement on the projection images can be directly translated into movements of the motors in virtual experiments. For future synchrotron experiments, a calibration between the pixel distance and motor movement needs to be performed to use the module. In addition, users can also intervene in the running alignment process simply by setting new values for the motors and overtake the algorithms, which can be more efficient when the samples are far away from the rotation center.

A mask module was introduced into the GUI to give the user an option to mask regions with high levels of impurities or where other samples may jump into the FoV and cause disruption. At the moment, the mask region is defined by user experience and needs further development to be more automatic.

## 4. Conclusion

In conclusion, for scanning tomography experiments, we present here automatic synchrotron tomographic alignment schemes based on genetic algorithms and human-in-the-loop software. These schemes are dedicated to addressing the needs to improve the sample alignment efficiency and automation in scanning tomography experiments and the necessary rotation-axis alignment prior to it. In terms of principle, our schemes divide each alignment job into two processes: coarse alignment and fine alignment. Coarse alignment is used for error correction on larger scales, while fine alignment allows for high-precision adjustments. This ensures accuracy while significantly reducing the time consumption on the overall process. Meanwhile, for the complex experimental conditions of scanning X-ray tomography, we have designed a GUI to supplement the algorithm, which provides sample detection, manual adjustment and sample monitoring functional modules as part of the coarse alignment workflow. Finally, the performance of our schemes was tested by virtual experiments; the misaligned rotation axis and sample were well aligned with sub-pixel accuracy when the effects of motor resolution and detector stability are excluded. The algorithms in each alignment step were encapsulated into a highly modular-designed software framework, which offers the possibility of future algorithm and functional upgrades. Looking to the future, we will also use a software approach to address the problems caused by the vibration and drift of the sample with respect to the beam which adds extra complexity for the alignment of the system. Together with the current work, a systematic software solution for the automation of tomographic alignment will be formed and will find wide applications in the data acquisition and online processing workflow of various synchrotron experiments.

The code for sample alignment is available from https://github.com/sampleAlign/sampleAlignment/tree/main/Code, and a GUI screen recording video of the sample alignment process can be obtained from https://github.com/sampleAlign/sampleAlignment/tree/main/GUI_Video.

## Supplementary Material

Supporting information file. DOI: 10.1107/S1600577522011067/tv5039sup1.pdf
