# Supplementary material for: Automatic synchrotron tomographic alignment schemes based on genetic algorithms and human-in-the-loop software
Source: J Synchrotron Radiat. 2023 Jan 1;30(Pt 1):169–78. doi: 10.1107/S1600577522011067 (PMC9814067; doi:10.1107/S1600577522011067)
Supplement: Supplementary file 1 [file s-30-00169-sup1.pdf]

JOURNAL OF SYNCHROTRON RADIATION

Volume 30 (2023)

Supporting information for article:

Automatic synchrotron tomographic alignment schemes based on genetic algorithms and human-in-the-loop software

Zhen Zhang, Xiaoxue Bi, Pengcheng Li, Chenglong Zhang, Yiming Yang, Yu Liu, Gang Chen, Yuhui Dong, Gongfa Liu and Yi Zhang

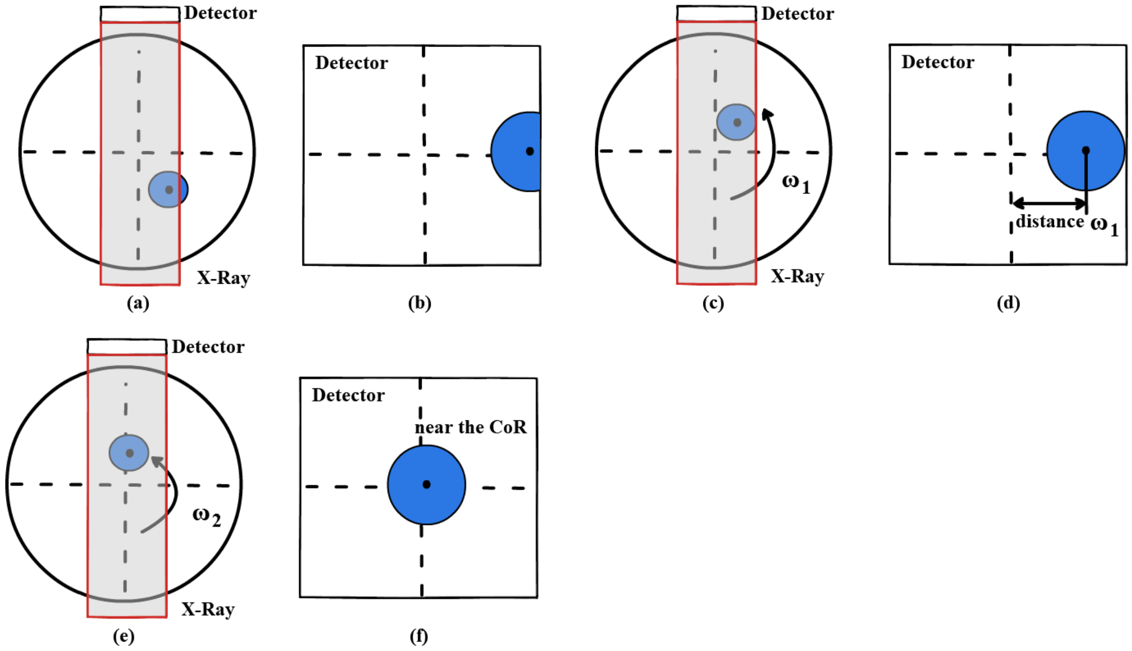

Figure S1 Schematic showing coarse sample alignment. A top view of the initial experiment setup is shown in (a) and the corresponding projection image (b), with the sample out of the FoV of the detector. Schematic showing the sample fully rotates (c,d) into the FoV of the detector. Schematic showing the sample rotates (e,f) into the center of FoV.

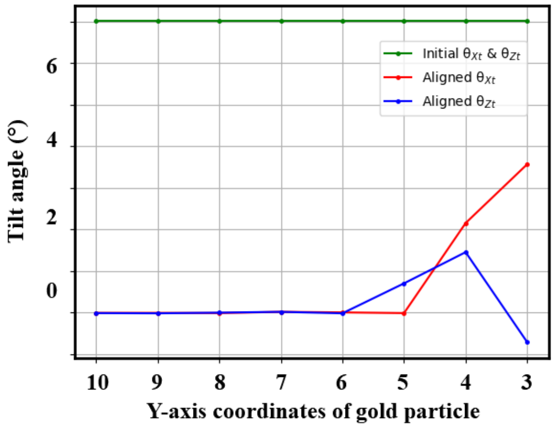

Figure S2 The effect of initial gold particle positions with respect to the rotation center for the alignment. The red line shows value of θ_{Xt} after each rotation axis alignment, while the blue line shows actual values of θ_{Zt}.
